# Efficacy of laparoscopic adenomyomectomy using double-flap method for diffuse uterine adenomyosis

**DOI:** 10.1186/s12905-015-0182-5

**Published:** 2015-03-13

**Authors:** Xiufeng Huang, Qiongshi Huang, Shuyi Chen, Jing Zhang, Kaiqing Lin, Xinmei Zhang

**Affiliations:** The Department of Gynecology, Women’s Hospital, Zhejiang University School of Medicine, 1 Xueshi Road, Hangzhou, Zhejiang 310006 P. R. China

**Keywords:** Adenomyosis, Adenomyomectomy, Dysmenorrhea, Double flap method, Surgery

## Abstract

**Background:**

Adenomyomectomy has recently been considered the priority option for the treatment of adenomyosis, however, the surgical efficacy and modes are still debated. We aimed to evaluate the efficacy of laparoscopic adenomyomectomy using a double-flap method for the treatment of uterine diffuse adenomyosis when compared with conventional laparoscopic adenomyomectomy.

**Methods:**

Laparoscopic adenomyomectomy using the conventional method (group A, *n =* 48) and the double-flap method (group B, *n =* 46) to treat diffuse uterine adenomyosis, respectively. Visual analog scale (VAS), menstrual amount, serum CA125 levels, and uterine volume were comparatively analyzed in both groups.

**Results:**

The VAS scores, menstrual amount, serum CA125 levels, and uterine volume at 12 or 24 months after surgery significantly reduced in group B than in group A (P < 0.05); these parameters were statistically decreased in both groups after surgery compared with those obtained before surgery (P < 0.001). Moreover, serum CA125 levels and uterine volume at six months of follow up were significantly lower in group B than in group A (P < 0.01). In addition, blood loss during surgery was similar in groups A and B (P > 0.05), although the operative time was significantly longer in group B than that in group A (P < 0.05).

**Conclusions:**

Laparoscopic adenomyomectomy using the double-flap method may be an effective technique to treat uterine diffuse adenomyosis.

## Background

Adenomyosis is a benign gynecologic disorder characterized by the invasion of endometrial glands and stroma in the uterine myometrium, resulting in dysmenorrhea, hypermenorrhea, and infertility [1]. Although adenomyosis may be treated with several methods, such as hysterectomy, conservative surgery, drug therapy such as gonadotropin-releasing hormone agonist therapy (GnRHa), and uterine artery embolization, complete hysterectomy can thoroughly treat this disease [2-9]. However, total hysterectomy is not suitable for women with adenomyosis who want to preserve their uteri and/or require fertility in the future. As such, these women prefer uterus-sparing surgery.

Although many uterus-sparing surgical techniques have been developed to treat adenomyosis, adenomyomectomy is considered as the most feasible and efficacious; adenomyomectomy has also been considered as the first-line approach to treat adenomyosis, particularly focal adenomyosis [10]. Partial adenomyomectomy including wedge resection of the uterine wall, transverse H incision technique, and asymmetric dissection of uterus to treat diffuse adenomyosis, can improve clinical symptoms; however, these techniques are frequently associated with adenomyosis recurrence and spontaneous uterine rupture in pregnancy [2,11-14]. The complete excision of adenomyosis by employing several techniques, such as overlapping flaps and triple-flap method to treat diffuse adenomyosis, can achieve good results; nevertheless, these techniques are difficult to implement, particularly laparoscopy [15-17]. These findings suggest that the development of a new surgical technique is a major concern to improve the convenience of laparoscopic conservative surgery to treat diffuse adenomyosis; with these novel techniques, adenomyotic lesions should be excised during surgery and uterine wall integrity should be retained.

Therefore, this study aimed to modify the technique of Osada et al. to perform laparoscopic adenomyomectomy by using a double-flap method for the treatment of uterine diffuse adenomyosis [16]. This study was also designed to evaluate the surgical efficacy of laparoscopic adenomyomectomy with the modified double-flap method for women with uterine diffuse adenomyosis, particularly those who manifested severe clinical symptoms and wished to preserve their uteri, but their reproductive capacity was not a priority request, compared with conventional laparoscopic adenomyomectomy.

## Methods

### Patients

The Ethics Committee of the Women’s Hospital, Zhejiang University School of Medicine approved this study. Written informed consent for participation in the study was obtained from participants.

A total of 129 patients who were referred to our hospital and underwent laparoscopic adenomyomectomy for uterine diffuse adenomyosis between March 2011 and February 2014 were recruited in this study. The inclusion criteria were listed as follows: ①women had severe dysmenorrhea with and without menorrhagia (hypermenorrhea), but failed to undergo drug therapy, including GnRHa, Mirena and oral contraceptives; ②women wished to preserve their uteri, but their reproductive capacity was not a priority request; ③Pure adenomyosis for all the study subjects was preoperatively verified by ultrasound and magnetic resonance imaging according to the previous reported diagnostic criteria [16-26], and affected more than 70% of the anterior and/or posterior wall of the uterus with an enlargement of >5 cm in thickness. The exclusion criteria were listed as follows: women presenting with a contraindication to laparoscopy because of severe medical illness. The patients who were recruited in this study were all interviewed by Dr. Huang. During her interview, each patient was in detail told about the advantage and disadvantage of the conventional method and the double-flap method (for example, less time and blood loss but less adenomyotic lesions excised may be in the former, whereas more time and blood loss but more adenomyotic lesions excised may be in the latter), and decide whether to participate in the study, and which method to take. Consquently, thirty-five among 129 patients who were invited to participate refused treatment. The 94 remaining patients with diffuse adenomyosis who were included in this study were assigned to undergo laparoscopic adenomyomectomy by using the conventional method (group A, *n* = 48) and the double-flap method (group B, *n =* 46) based on patient requirements. After surgery was completed, all of the patients received GnRHa for six months. None of the study patients revoked their consent, failed to undergo follow up, or received sex-hormone therapy six months before surgery.

### Surgical procedure

All surgical procedures were performed under general anesthesia in the Trendelenburg position with four-port laparoscopy. One 10 mm port was inserted through the umbilicus for the zero-degree laparoscope, and two lateral 5 mm ports were inserted above and medial to each anterior superior iliac spine. A second left sided 5 mm port was inserted between the left lateral port and the umbilical port. The surgeon (XZ and XH) used the two left sided ports to perform most of the surgical procedures.

The technique of resection of adenomyotic lesions using the double-flap method was previously described by Osada et al. [16]. In brief, 12 units of pituitrin (diluted in 100 ml of normal saline) were injected. An incision was made in the midline of the serosal surface of the fundus by using scissors (or monopolar) and continued along the sagittal direction until the uterine cavity was reached. The incision was further continued along the posterior and anterior walls of the uterus to the level of the internal os of the cervix. Afterward, adenomyomatous tissues were grasped with forceps, identified, and excised from the surrounding myometrium. This procedure was performed with care to avoid damaging the endometrium and the serosal surface of the uterus. If the myometrium appeared normal, this part was spared as much as possible. In general, a myometrial thickness of 1 cm below the serosa or above the endometrium was left. In addition, this procedure was performed with care to avoid damaging the interstitial portion of the fallopian tube, particularly in patients who desired to have future pregnancies.

**Figure 1:**
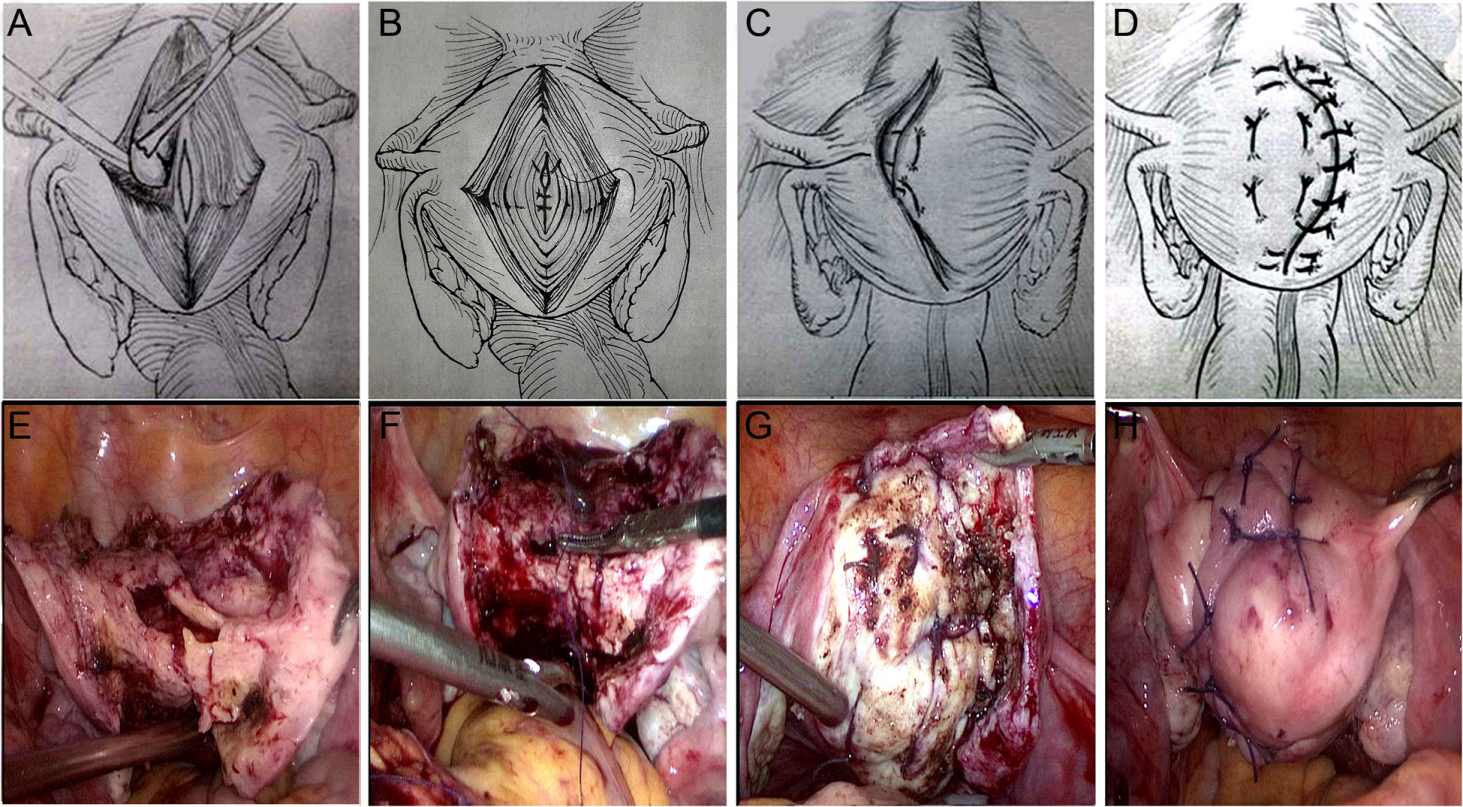
**Schematic (A, B, C, and D) and surgical view (E, F, G, and H) of laparoscopic adenomyomectomy using the double-flap method. (A and E)** after complete removal of adenomyotic lesions using the resection technique of Osada et al. **(B and F)** closure and reconstruction of the uterine cavity using 3–0 absorbable suture. **(C and G)** the first flap in one side wall of the uterus is brought into the second flap in another side of the uterine wall such that the other side wall of the uterus is covered. **(D and H)** the second flap in another side of the uterine wall is brought to cover the first flap in one side wall of the uterus (before overlapping occurs, the serosal surface of the underlying flaps is stripped to ensure that only myometrial tissue flaps are overlapped).

**Figure 2:**
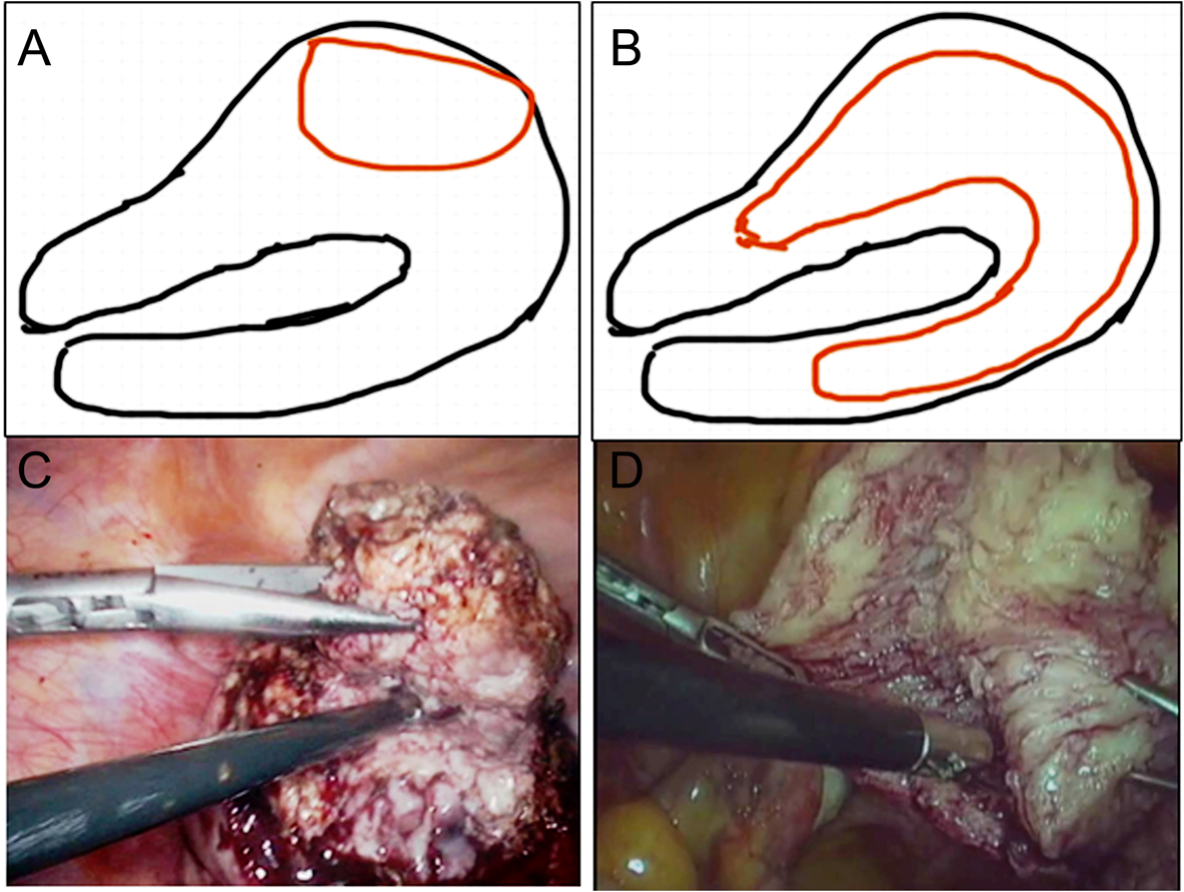
**Comparisons of surgical view and schematic of laparoscopic adneomyomectomy using the double-flap method and the conventional method. (A, C)** Conventional method; **(B, D)** double-flap method.

After adenomyotic lesions were removed (Figure 1A and E), the endometrial lining was approximated with interrupted sutures of 3–0 Vicryl (Figure 1B and F). The myometrium and serosa of the bisected uterus were sutured with 2–0 Vicryl by using the double-flap method described by Kim et al. [17], but not by using the triple-flap method proposed by Osada et al. [16]. Namely, the first flap in one side wall of the uterus (including the serosa and the myometrium) was brought into the second flap in another side of the uterine wall (including the endometrium and the myometrium) such that the other side wall of the uterus (including the endometrium and the myometrium) was covered (Figure 1C and G). Next, the second flap in another side of the uterine wall was brought to cover the first flap in one side wall of the uterus (Figure 1D and H). Before overlapping occurred, the serosal surface of the underlying flaps was stripped to ensure that only myometrial tissue flaps overlapped. During the suture procedure, dead space or hematoma between the tissues was avoided. The conventional surgical procedure was similar to that of myomectomy and completely different from the new surgical procedure (Figure 2). After the surgical procedure, we used INTERCEED (an anti adhesion membrane, Johnson company) to prevent postoperative adhesion. All excised adenomyotic tissues were confirmed by histopathology after surgery.

### Follow up

Surgical efficacy was evaluated by rating the levels of serum CA125, the size of the uterus, and the severity of dysmenorrhea and hypermenorrhea before and after surgery as well as the presence of pregnancy after surgery in the two groups. The severity of dysmenorrhea was documented using a standardized questionnaire with a visual analog scale (VAS). The pain scale was subdivided into ten grades. “No pain” was indicated at the left side of the scale and “the maximum pain you could imagine” was designated at the right side of the scale. The size of the uterus was measured by ultrasonography [uterine volume = *A* × *B* × *C* × 0.5233 (where *A*, *B*, and *C* are the uterine length, width, and thickness, respectively)]. Serum CA125 levels were determined by enzyme-linked immunosorbent assay (ELISA) with a human CA125 ELISA kit (HM10776, Bio-swamp) according to the manufacturer’s instructions (the normal range was ≤35 kU/L). The menstrual product use of ≥5 pads/day was defined as menorrhagia in this study; as such, the severity of menorrhagia was arbitrarily graded as mild (5 to 7 pads/day), moderate (7 to 9 pads/day), and severe (>9 pads/day) [27].

All of the patients were followed up one, three, and six months after surgery and subsequently every six months after surgery. Considering that these patients were treated with GnRHa for six months after surgery, we focused on two endpoints to determine short- and long-term surgical treatment efficacies. As such, the results of the preoperative visit were compared with those of the 12-month follow up and those of the 24-month follow up to observe the short- and long-term efficacies after laparoscopic adenomyomectomy was performed using the double-flap method and the conventional method to treat uterine diffuse adenomyosis.

### Statistical analysis

We used SPSS version 17.0 (SPSS, IBM, Chicago, IL, USA) to perform statistical analyses. Results were expressed as the mean value ± standard derivation (SD), although the measured values of the variables were not normally distributed. Mann–Whitney *U* test was performed to compare non-parametric data, and chi-square test was conducted to compare the frequency between groups. Differences were considered significant at P < 0.05.

## Results

**Figure 3:**
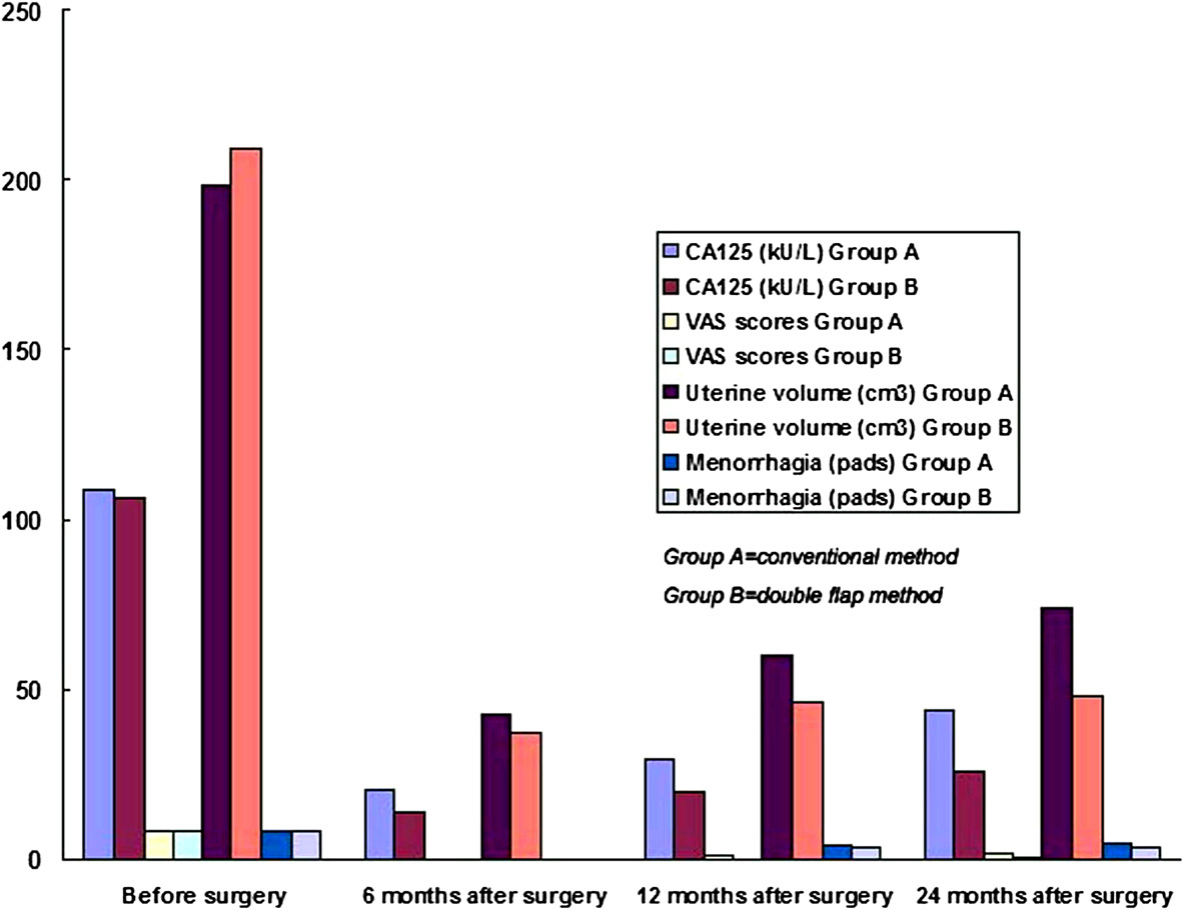
**Changes of serum CA125 levels, uterine size, pain scores, and menorrhagia before and after surgery in groups A and B.** Group **A** = Conventional method, Group **B** = Double-flap method.

No significant differences in age, gravidity, parity, abortion, hemoglobin levels, uterine volume, VAS score, menorrhagia, and serum CA125 levels were found between the two groups (P > 0.05, Table 1). Six months after surgery, five patients (5/48, 10.4%) in group A and two patients (2/46, 4.3%) in group B still exhibited pain symptoms with VAS scores of ≤2. The VAS scores at six-month follow-up period in group A or B significantly decreased compared with those before surgery (P <0.0001), but no statistically significant differences were found between groups A and B (P > 0.05, Table 2, Figure 3). Uterine size and serum CA125 levels six months after surgery were significantly higher in group A than in group B (P < 0.0001), although both parameters in each group were statistically decreased compared with those obtained before surgery (P <0.0001, Table 2, Figure 3).

**Table 1:** **Patients’ characteristics (Mean ± SD)**

**Parameters**	Group A* ( ***n =*** **48)**	**Group B (** ***n =*** **46)**	**P value**
Age at operation(years)	36.6 ± 5.9	37.1 ± 6.6	0.187
Parity	1.1 ± 0.1	1.1 ± 0.1	0.321
Gravidity	3.4 ± 0.2	3.5 ± 0.2	0.165
Abortion	2.3 ± 0.2	2.4 ± 0.2	0.245
Hemoglobin (g/dl)	10.6 ± 2.2	10.8 ± 2.3	0.209
CA125 (kU/L)	108.7 ± 168.9	106.5 ± 199.5	0.654
VAS score	8.1 ± 1.6	8.2 ± 1.5	0.197
Uterine volume (cm^3^)	198.5 ± 82.6	209.1 ± 117.5	0.346
Menorrhagia (pads)	8.2 ± 1.5	8.1 ± 1.3	0.278

*Group A = Conventional method, Group B = Double-flap method.

**Table 2:** **Changes in serum CA125 levels, uterine size, pain scores, and menorrhagia after surgery in groups A and B**

**Parameters**	**Serum CA125**	**Pain scores**	**Uterine size**	**Menorrhagia**
	**(kU/L)**		**(cm** ^**3**^ **)**	**(pads)**
Group A* (*n =* 48)				
6 months* (*n =* 48)	20.3 ± 6.9	0.2 ± 0.5	43.0 ± 12.1	------
12 months (*n =* 31)	29.4 ± 18.3	0.8 ± 1.1	59.7 ± 24.1	4.2 ± 0.9
24 months (*n =* 15)	43.8 ± 20.7	2.0 ± 2.1	74.0 ± 30.6	4.6 ± 1.1
Group B (*n =* 46)				
6 months (*n =* 46)	13.3 ± 3.9	0.1 ± 0.3	37.6 ± 4.6	------
12 months (*n =* 27)	19.7 ± 6.2	0.2 ± 0.6	45.8 ± 4.9	3.7 ± 0.6
24 months (*n =* 13)	25.6 ± 6.7	0.4 ± 0.9	48.1 ± 5.1	3.8 ± 0.6

*Group A = Conventional method, Group B = Double-flap method.

Twelve months after surgery, nine patients (9/31, 29.0%) in group A and three patients (3/27, 11.1%) in group B manifested pain symptoms, and the VAS scores were ≤3.0. In group A, the VAS scores increased at 12 months after surgery compared with those at 6 months after surgery (P <0.01); the VAS scores increased at 24 months after surgery compared with those at 12 months after surgery (P <0.05). In group B, the VAS scores were similar between 12 and 6 months after surgery and between 24 and 12 months after surgery (P > 0.05). The VAS scores at 12 and 24 months after surgery were significantly higher in group A than in group B (P < 0.05); these scores in each group were significantly lower compared with those before surgery (P < 0.0001, Table 2, Figure 3). Twenty-four months after surgery, nine patients (9/15, 60.0%) in group A reported pain, and their highest VAS score was 5.5; by contrast, three patients (3/13, 23.1%) in group B reported pain, and their VAS scores were ≤2.5.

Uterine volume significantly increased at 12 months after surgery compared with those at 6 months after surgery (P < 0.0001). Furthermore, the uterine volume of the two groups significantly increased at 24 months after surgery compared with those at 12 months after surgery (P < 0.01, Table 2, Figure 3). Uterine sizes at 12 and 24 months after surgery were statistically higher in group A than in group B (P < 0.0001); uterine size in each group was significantly decreased after surgery compared with that before surgery (P < 0.0001, Table 2, Figure 3). Similar to uterine size, serum CA125 levels at 12 and 24 months after surgery were significantly higher in group A than in group B (P < 0.05); serum CA125 levels were also statistically lower than those before surgery (P = <0.0001, Tables 1 and 2). Differences in serum CA125 levels between 6 and 12 months and between 12 and 24 months after surgery were significantly different in both groups A and group B (P <0.05). In group B, all of the patients revealed normal serum CA125 levels at any month after surgery; by contrast, the serum CA125 levels of six (19.4%) patients in group A at 12 months and seven (46.7%) patients at 24 months after surgery were >35 kU/L.

The numbers of healthy pads used during menstruation at 12 and 24 months after surgery were significantly higher in group A than in group B (P <0.05), but the number of pads in each group was significantly lower than that before surgery (P < 0.0001, Table 2, Figure 3). The differences in menstrual flow between 12 and 24 months after surgery were not statistically significant in either of the groups (P > 0.05), although the menstrual flow at 24 months after surgery increased compared with that at 12 months after surgery in both groups (Table 2, Figure 3). In group B, all of the patients experienced normal menstruation after surgery. In group A, six (19.4%) patients at 12 months and five (33.3%) patients at 24 months after surgery suffered from menorrhagia, but the number of pads used was ≤7.

The amount of blood loss during surgery was similar in groups A and B (137.5 ± 54.6 ml vs. 145.6 ± 61.6 ml, P > 0.05). Accordingly, the amount of hemoglobin loss between before and after surgery was also similar in groups A and B (0.5 ± 0.26 g/dl vs. 0.6 ± 0.37 g/dl, P > 0.05). The operative time was much more in group B (152.5 ± 106.9 min) than that in group A (116.7 ± 53.8 min, P < 0.05). Next, the weight of the excised tissues was heavier in group B than that in group A (177 ± 155 g vs. 235.7 ± 201.3 g, P < 0.05). Furthermore, no intraoperative or postoperative complications were found in groups A and B. In addition, ten patients after surgery in this study (group A = 6, group B = 4) who wished to have future pregnancies did not want to have a pregnancy so far, because they have had children.

## Discussion

The present results showed that all of the study patients exhibited a significant reduction in pain symptoms, menorrhagia, serum CA125 levels and uterine size after surgery. Dysmenorrhea and menorrhagia are the characteristic symptoms of adenomyosis, and directly related to the surgical efficacy of laparoscopic adenomyomectomy [9,28,29]. Serum CA125 levels are considered as a good biomarker to diagnose and monitor the therapeutic efficacy and recurrence of adenomyosis [30,31]. An enlarged uterus is also a major symptom of adenomyosis, and a reduction in uterine size is also directly associated with therapeutic efficacy [32,33]. It is apparent that laparoscopic adenomyomectomy can treat diffuse uterine adenomyosis effectively [4,10,34-36]. However, menorrhagia, serum CA125 levels and uterine size were increased after surgery when postoperative follow up was prolonged even if we used GnRHa therapy for six months after operation. It is indicated that the surgical efficacy of laparoscopic adenomyomectomy for the treatment of diffuse uterine adenomyosis can decrease over time. Therefore, long-term drug therapy such as Mirena (or oral contraceptives) is recommeneded after adenomyomectomy for the treatment of diffuse uterine adenomyosis [17,37].

In fact, the surgical efficacy of adenomyomectomy is dependent on the type and extent of adenomyosis as well as the modes of surgery [10,37,38]. Theoretically, adenomyomectomy can achieve good results for focal adenomyosis (or adenomyoma), but not for diffuse adenomyosis. Complete resection of adenomyotic lesions (type I) can have more surgical efficacy compared with cytoreductive surgery of adenomyosis (type II) [10,37]. In our study, the triple-flap method was modified by changing the mode of surgery and the suturing method, but the resection method was retained [16]. Obviously, the double-flap method is classified as type I, while the conventional method is classified as type II [10,37]. Our results showed that the VAS scores, the number of healthy pads, serum CA125 levels and uterine volume at 12 or 24 months after surgery were all significantly lower when the double-flap method was used than when the conventional method was used. Moreover, all of the patients experienced normal CA125 levels and menstruation, and the VAS scores were similar after surgery when the double-flap method was used. By contrast, 25% patients still suffered from menorrhagia, about half of patients showed high serum CA125 levels, and the VAS scores increased after surgery when the conventional method was used as follow up time was prolonged. These results indicate that laparoscopic adenomyomectomy using the double-flap method was more effective to treat uterine diffuse adenomyosis than conventional laparoscopic adenomyomectomy, which are similar to the previous reports [10,37].

Recently, Saremi et al. performed open wedge-shaped adenomyomectomy for 103 patients with adenomyosis, and 21 (30%) out of 70 patients who attempted pregnancy achieved a clinical pregnancy [39]. Kishi et al. treated 102 patients with adenomyosis who had a desire for pregnancy by laparoscopic adenomyomectomy using the conventional method, and the clinical pregnancy rates in women with age ≤39 years and ≥ 40 year were 41.3% and 3.7%, respectively [40]. In our study, 10 patients who wished to conceive after surgery did not want to have a pregnancy so far, because their age were relatively older, and they have had children, which is in agreement with the study of Kim et al. [17]. Actually, our study and the study of Kim et al. contain less infertility patients compared with the studies of Kishi et al. and Saremi et al. [17,39,40]. Moreover, patients with age >40 years do not show a clear benefit of the surgery on fertility outcomes after adenomyomectomy for the treatment of adenomyosis [39]. Furthermore, in patients with extremely severe diffuse adenomyosis, it is quite difficult to maintain the intact morphological and functional reconstruction after complete removal of adenomyotic lesions. In such cases, it is hard to tell patients whether they have a future pregnancy [39]. Therefore, the fear of future pregnant uterine rupture may also be a factor for our patients with severe diffuse adenomyosis who do not want to have a pregnancy at present [41].

Although Kim et al. reconstructed the uterine wall using the double flap method after laparoscopic-assisted adenomyomectomy, yet, their resection method quite differs from our resection method [17]. As matter of fact, we initially try to perform open adenomyomectomy by using the technique of Osada et al. [16]. When we find it is extremely difficult to reconstruct the uterine wall using the triple-flap method in despite of a new absorbable barbed suture (v-loc) [16,37], then we try to suture using the double flap method. After we have mastered the technique of the double flap method, we perform a laparoscopic surgery. During the surgical procedure, a diluted solution of pituitrin was first injected until the uterus became white colour, and then the remained pituitrin solution was continually used when it was needed. In the meantime, a drainage tube used as a tourniquet for transient occlusion of uterine arteries was placed into the abdominal cavity in case of massive bleeding during the procedure [42]. Moreover, the uterine cavity was opened so that the entire extent of the adenomyosis, the crucial landmarks of the endometrium and the serosal surface are clearly visible [16]. We found no patients required conversion to open surgery, and the blood loss was similar in the two methods, although the double-flap method had more operative time compared with the conventional method. Interestingly, the amount of the blood loss in our study (145.6 ml) is less compared with the study of Kim et al. (383.3 ml), while the operative time is a little longer in our study (152.5 min) in comparison with the study of Kim et al. (130.6 min) [17]. Moreover, no intraoperative or postoperative complications were observed in all of the study patients. In addition, complete removal of the adenomyotic lesions may create better uterine conditions for pregnancy [16]. Therefore, the double-flap method could be safe and effective to treat uterine diffuse adenomyosis, although future follow-up observation is needed for postoperative pregnancy and childbirth in order to verify the robustness of the uterine reconstruction.

## Conclusions

Our results showed that women with diffuse adenomyosis exhibited a significant reduction in serum CA125 levels, uterine size, hypermenorrhea, and dysmenorrhea after laparoscopic adenomyomectomy was performed using the double-flap method. These results suggest that adenomyomectomy with the double-flap method may be a good therapeutic option for women with diffuse uterine adenomyosis and wish to avoid hysterectomy. Nevertheless, further studies should be conducted to verify these results.
